# Institutional Design and Incentives for Migrant Workers to Participate in Social Insurance in China: Evidence From a Policy Experiment in Chengdu City

**DOI:** 10.3389/fpubh.2021.736340

**Published:** 2021-10-21

**Authors:** Yihao Tian, Yuxiao Chen, Mei Zhou, Shaoyang Zhao

**Affiliations:** ^1^Department of Public Service Management and Public Policy, School of Public Administration, Sichuan University, Chengdu, China; ^2^Department of Public Administration, School of Politics and Public Administration, Zhengzhou University, Zhengzhou, China; ^3^Department of Social Security, School of Insurance, Southwestern University of Finance and Economics, Chengdu, China; ^4^Department of Finance, School of Economics, Sichuan University, Chengdu, China

**Keywords:** social insurance, contribution rate, participation incentive, migrant workers, difference-in-difference

## Abstract

Rural-to-urban migration has increased rapidly in China since the early 1980s, with the number of migrants has reached 376 million by 2020. Despite this sharp trend and the significant contributions that migrants have made to urban development, the migrant workers have had very limited access to the social insurance that the majority of urban workers enjoy. Against the background of the social insurance system adjustment in Chengdu in 2011, this study uses a difference-in-differences (DID) model to empirically test the impacts of changes in the social insurance policy contribution rates on the social insurance participation rates of migrant workers, using the China Migrants Dynamic Survey (CMDS) data for 2009–2016. We find that the social insurance participation rate of migrant workers was significantly reduced after they were incorporated into the urban worker insurance system. There was no significant change in the wages of migrant workers, but the working hours were increased and their consumption level decreased. In other words, simply changing the social insurance model of migrant workers from “comprehensive social insurance” to “urban employee insurance” reduces the incentives for migrant workers to participate in insurance and harms the overall welfare of migrant workers. Our study indicates that the design of the social security policy is an important reason for the lower participation rate of migrants. It is necessary to solve the problem of insufficient incentives through the targeted social security policies; primarily, the formulation of a social security policy contribution rate suitable for the migrants, and the establishment of a comprehensive social security policy and the gradual integration of the social security system.

## Introduction

**Figure 1:**
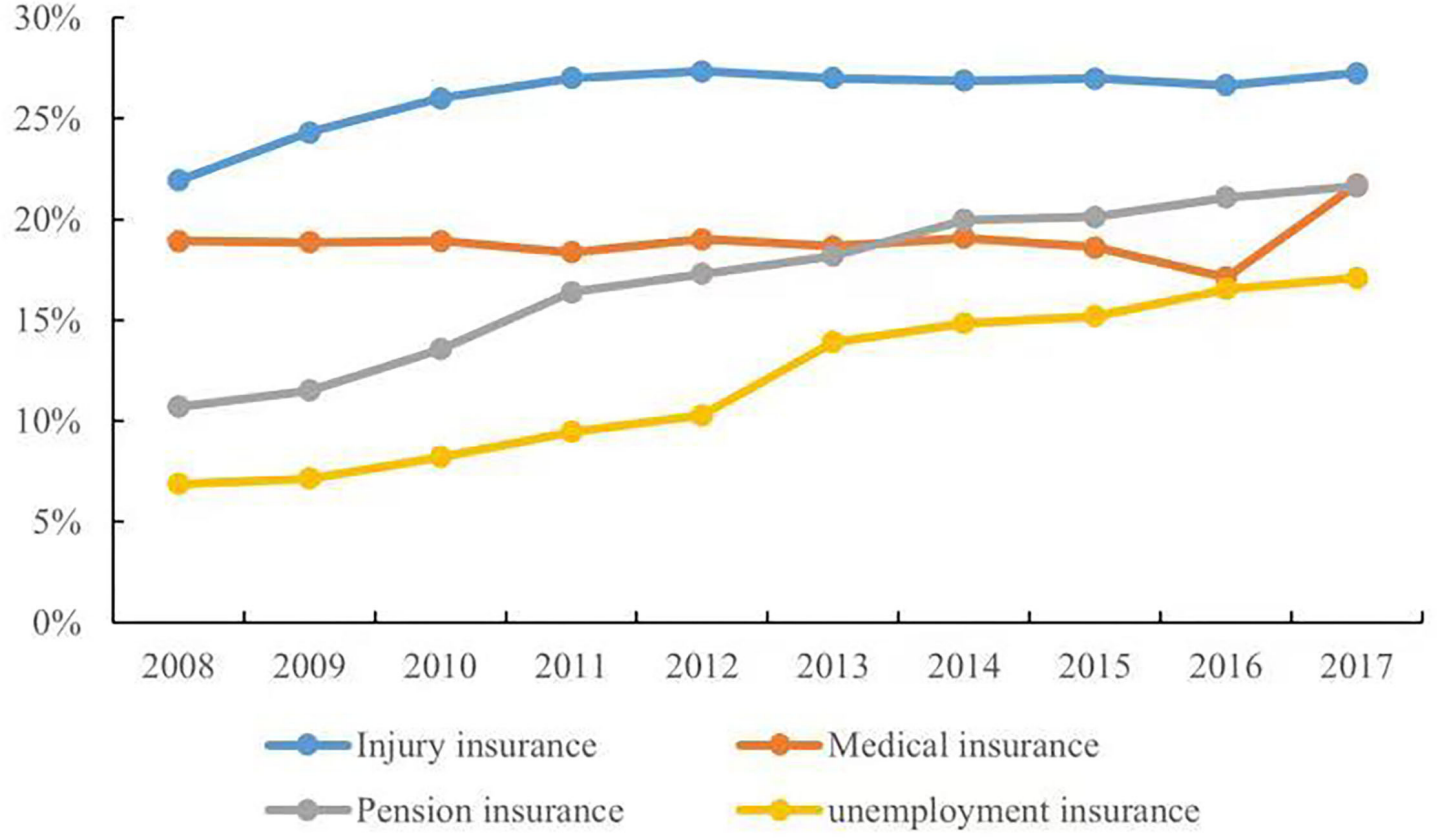
The proportion of migrant workers participating in the urban basic social insurance. Data obtained from the Bulletin of Human Resources and Social Security Development Statistics (2008–2017).

In recent years, the social insurance system of China has gradually improved, and the social insurance coverage rate for urban workers had reached 54.01% by 2018. However, the social insurance coverage for migrant workers is still far behind that of other groups. Figure 1 depicts the participation of migrant workers in various insurance types. The number of migrant workers participating in social insurance has increased year by year, but the participation rate in pension and medical insurance is around 20%, while the participation rate in unemployment insurance is only 15%, and the highest participation rate, in injury insurance, is less than 30%. According to the Seventh Census of China, taken in 2020, the number of migrant workers had reached 376 million, accounting for 26.63% of the total population. The migrant workers have made great contributions to urban development, but they do not enjoy the same benefits as the local urban workers (1–4). In China, social insurance is often tied to household registration called hukou; however, the places where they work, are separate from the location of their household registrations. As a result, migrant workers are often excluded from social insurance coverage in inflow cities. Given a large number of migrant workers, participation in basic social insurance in inflow cities is crucial to the smooth urban integration of migrant workers. The general lack of protection for the migrant workers in terms of work injury, unemployment, medical care, and pension will undermine their labor rights and interests, and weaken their resistance to risks, while also increasing social inequality (5–7).

The three causes of this low participation rate have been summarized in the literature. First, the social insurance system for migrant workers in China has poor portability and fragmentation (2). The migrant workers suffer from institutional discrimination in the job market and have difficulty participating in the local social insurance in the inflow cities. In addition, it is difficult to transfer social insurance; for example, the medical insurance purchased by migrant workers in their home location cannot be used in the inflow city (2, 8–11). Second, the demand characteristics of migrant workers are an important reason for their reduced willingness to participate in insurance. The migrant workers, who are relatively less educated, underestimate their risk of illness and lack sufficient far-sightedness to consider the issue of securing a pension for the future. Given the low income of migrant workers, they often choose to increase their current income and give up participating in social security, in the face of a high social insurance premium rate (12, 13). Third, the social insurance for employees is paid by both the firm and the employee in China, with the firm contributing about 80% of the premium. The social insurance cost of employees already accounts for more than 40% of the labor cost of firms in China, which is significantly higher than that in other Asian countries (14). Most firms that employ migrant workers are labor-intensive firms with relatively low-profit margins. The social insurance costs increase the operating burden on the firms. Consequently, many of these firms choose not to provide insurance for their employees (15–21).

With the improvement of the social insurance system, the institutional barriers faced by the migrant workers to participate in social insurance have gradually been eliminated. However, the problem of insurance evasion by individuals and enterprises due to insufficient incentives for social insurance participation has become increasingly prominent. For the special group of migrant workers, what kinds of trade-offs will they make when facing the costs and benefits of different social insurance patterns? Which would they prefer, a low premium rate and a low social insurance benefit or a high premium rate and a high social insurance benefit? These questions have not been answered well. Given the low participation rate of migrant workers in social insurance, it is necessary to discuss the incentive problem of participation in social insurance from the perspective of social insurance pattern design. Only by considering the particularity of migrant workers and providing sufficient incentives to them to participate in insurance, the coverage of social insurance can be effectively expanded. The insurance incentives for the migrant workers are more flexible, especially when the setting of the policy premium rate is more sensitive, which needs further consideration.

At present, there are two main patterns of social insurance for migrant workers. One is the Urban Employee Basic Medical Insurance (UEBMI), which includes basic pension insurance, basic medical insurance, unemployment insurance, work injury insurance, and maternity insurance; most enterprises will buy complete insurance packages for their employees, but some enterprises only buy some types of insurance to reduce the costs. Comprehensive insurance (CI) is the other type of insurance developed for migrant workers. CI features some important insurance policies, such as work injury insurance and medical insurance. Compared with UEBMI, CI has a lower level of benefits, but the premium rate is also lower as shown in Table 1.

**Table 1:** Comparison of the two social insurance models.

**Social insurance patterns**	**Premium rate**	**Social insurance benefit**
Urban Employee Basic Medical Insurance	High	High
Comprehensive Insurance	Low	Low

Chengdu, the central city in western China, absorbs a large number of migrant workers. According to the data of Chengdu's Seventh Census, taken in 2020, the number of migrant workers has reached 10.72 million, ranking among the top 10 in China. The social insurance pattern of the migrant workers in Chengdu saw a significant shift from CI to UEBMI after 2011, providing a quasi-natural experimental environment to answer these questions. Up to 2011, Chengdu had provided CI for the migrant workers, which included five types of insurance, such as pension insurance and basic medical insurance. The premium for comprehensive reimbursement was 20% of the premium base, of which 14.5% was borne by the enterprises and 5.5% by the migrant workers. After 2011, Chengdu provided UEBMI for migrant workers, and although the insurance benefits were higher, it also increased the burden of payment for both the firms and migrant workers. This reform did not include migrant workers in the construction industry, who continued to be insured by CI, thus providing a control group for this study. Based on the China Migration Dynamics Survey data for 2009–2016, this study uses the difference-in-differences (DID) method to empirically verify the impact of social insurance policy premium rate setting on the insurance participation of migration workers in Chengdu. We attempt to provide a strong complement to analyze the equity of social insurance participation and incentives to participate in the insurance for migrant workers in China.

## Methodology

### Data Source

This study used the 2009–2016 data from the China Migrants Dynamic Survey (CMDS), conducted by the National Health Commission, P.R. China. The CMDS subjects are migrant workers from 31 provinces, autonomous regions, municipalities, and Xinjiang Production and Construction Corps, aged 16–59, with agricultural household registrations, and working as employees. These subjects have lived in the place of inflow cities for more than 1 month and do not have local household registration (Hukou). The survey has been conducted annually since 2009 but does not comprise balanced panel data because the sample is not the same from year to year. The data have a long-term span, strong representativeness, and a rich set of variables. The survey not only encompasses the basic characteristics of migrants, but also their work, family members, and insurance participation.

### Variables

Participation by migrant workers in injury insurance is the highest and most practical, so this study mainly examines the participation rate in the injury insurance of workers. The key dependent variable in our analysis relates to the decision to participate in injury insurance. Three indicators were utilized: first, whether to participate in injury insurance; second, whether to participate in either injury insurance or medical insurance; and third, whether to participate in any one of injury insurance, medical insurance, or pension insurance. In addition, this study verified the effects of premium rates on the wages, work hours, and consumption of migrant workers.

In the regression analysis, the study also controlled for the variables of the nature of migrant workers' employment units, working years, gender, age, education level, and the number of family members. The nature of the employment units is a dummy variable, which is equal to (1) if the unit of a migrant worker is a state-owned firm, (2) if it is a private firm, (3) if it is a mixed operation firm, and (4) if it is of another nature. The working years refer to the duration migrant workers have worked for since they left their hometowns.

### Descriptive Statistics

As shown in Table 2, although the social insurance participation rate of the migrant workers has increased over the years, the overall level of participation in insurance was still very low. The participation rates in pension and medical insurance are about 20%, participation in unemployment insurance is less than 20%, and even the highest participation rate, in injury insurance, only averages about 30%. The numbers for maternity insurance and housing provident fund are even lower. The proportion of migrant workers participating in the UEBMI is still relatively low.

**Table 2:** The participation of migrant workers in social insurance from 2009 to 2016.

**Year**	**Pension**	**Medical insurance**	**Unemployment insurance**	**Injury insurance**	**Maternity Insurance**	**Housing provident fund**
2009	0.259	0.276	0.096	0.461	0.062	0.019
2010	0.125	0.053	0.101	0.287	0.071	0.033
2011	0.229	0.269	0.141	0.313	0.103	0.052
2012	-	0.276	-	-	-	-
2013	0.223	0.214	0.185	0.321	0.069	0.066
2014	0.241	0.228	0.191	0.276	0.138	0.083
2015	-	0.254	-	-	-	-
2016	0.311	0.224	0.274	0.333	0.237	0.123

As shown in Table 3, the migrant workers are mainly engaged in manufacturing, construction, wholesale and retail, catering services, and social service industries. These industries often provide jobs with high labor intensity, low wages, high potential risks, and strong alternatives. Table 4 lists the social insurance coverage in different industries based on the 2016 survey data, such as five types of insurance and the housing provident fund. There are two obvious features. First, there are huge differences in social insurance coverage among the industries. The industries with the highest coverage rate of nearly 50% include mining, electricity, coal, water and heat supply, financial insurance, and real estate. However, there are very few migrant workers in these industries (as shown in sample proportion); the relatively low coverage rate of about 10% is found in agriculture, forestry, animal husbandry, fishing, construction, accommodation, catering, and others, and the migrant workers in these industries account for a larger proportion of the workforce. Second, the coverage rates of the five insurances and the housing provident fund are almost the same in different industries. That is, the companies either provide five kinds of insurance and the housing provident fund at the same time or do not provide them at all. Comparatively speaking, the coverage rate of provident funds is the lowest, and work injury insurance is the highest among the five insurances, followed by pension, unemployment, and medical insurance.

**Table 3:** Industry distribution of migrant workers (%).

**Industry**	**2009**	**2010**	**2011**	**2012**	**2013**	**2014**	**2015**	**2016**
Agriculture	0.31	1.85	2.03	2.29	2.22	2.64	1.95	2.32
Mining	0.37	2.23	2.12	1.76	1.61	1.71	2.12	1.42
Manufacturing	44.34	35.88	36.50	34.64	34.37	31.40	35.61	30.18
Electric, coal and water production and supply	0.48	0.71	0.67	0.77	0.77	0.83	0.58	0.68
Construction	7.67	12.49	14.07	12.51	11.68	11.29	9.61	10.25
Transportation	3.24	4.44	4.35	4.36	4.00	4.31	4.04	4.92
Wholesale and retail	8.80	7.05	6.78	7.91	7.88	7.78	10.74	9.66
Accommodation	11.85	13.51	11.49	12.89	14.07	14.55	11.65	12.73
Financial, Insurance and Real Estate	1.06	0.83	1.22	1.31	1.42	2.05	1.93	3.12
Social service	15.52	14.47	11.32	10.84	11.35	18.66	16.81	18.62
Public administration	0.90	3.02	2.90	3.38	3.76	4.77	4.96	6.09
others	5.45	3.52	6.57	7.33	6.88	0.00	0.00	0.00

**Table 4:** Social insurance coverage of migrant workers by industry in 2016.

**Year**	**Pension**	**Medical insurance**	**Unemployment insurance**	**Injury insurance**	**Maternity Insurance**	**Housing provident fund**
Agriculture	0.136	0.064	0.077	0.105	0.073	0.037
Mining	0.544	0.345	0.472	0.650	0.279	0.297
Manufacturing	0.426	0.310	0.386	0.504	0.331	0.157
Electric, coal and water production and supply	0.541	0.384	0.444	0.553	0.377	0.300
Construction	0.114	0.073	0.090	0.181	0.076	0.035
Transportation	0.306	0.234	0.268	0.299	0.217	0.113
Wholesale and retail	0.247	0.182	0.215	0.221	0.198	0.081
Accommodation	0.159	0.105	0.128	0.145	0.107	0.041
Financial, Insurance and Real Estate	0.438	0.338	0.400	0.442	0.371	0.244
Social service	0.292	0.205	0.261	0.281	0.229	0.127
Public administration	0.473	0.375	0.425	0.443	0.395	0.247

### Estimation Models

To study whether an increase in the contribution rate of social security policies will reduce the incentives for migrant workers to participate in insurance, it is necessary to compare the changes in the participation of migrant workers in social security before and after the increase. However, other factors affecting the participation of migrant workers in social security also changed during the period under examination. For example, the implementation of the new Social Insurance Law in 2011 had a huge impact on the participation of migrant workers. Therefore, it is necessary to introduce the DID model, which is very suitable for assessing the policy effects. The specific method for constructing the DID model involves the establishment of a “treatment group,” which has experienced an increase in the social security contributions, and a “control group,” which has not undergone this change. By controlling other factors, comparisons can be made between the treatment group and the control group to test the effects of policy implementation. Chengdu's unique social security policy changes regarding the migrant population provide a good opportunity to build a DID model. The transformation of the social security system of non-urban employees in Chengdu was very different for those in the construction companies and those in other companies. First, those in non-construction enterprises experienced a significant change from CI to UEBMI in 2011, and their social security contribution ratio greatly increased; this can be regarded as the treatment group. The policy for non-urban household registration employees in construction enterprises remained unchanged; however, their social security contribution ratio was stable at 4%. They can, thus, be regarded as the control group. We use the differences in the social security policies of different industries in Chengdu around 2011 to construct the DID model:


(1) $$\begin{array}{l} Insurance_{\text{it}}=\beta_{0}+\beta_{1}{Time}_{\text{it}}+\beta_{2}Industry_{\text{it}}+\beta_{3}Industry_{\text{it}}\\ \;*Time_{\text{it}}+X_{it}\gamma +\varepsilon_{it} \end{array}$$

In this equation, *Insurance* represents whether the migrant participates in the basic social insurance. This can be divided into three categories according to the importance and practicability of the insurance: participation in work injury insurance, participation in a work injury or medical insurance, and participation in a work injury, medical, or pension insurance. *Time* represents the time of the policy change, which is set to 1 in 2011 and thereafter, and 0 otherwise. *The industry* represents the industry in which the migrant workers are located and is divided into four categories according to the nature and scale of the work: “construction,” “wholesale and retail, and accommodation, and catering,” “manufacturing,” and “other industries.” To test the effects of policy reform, we set the cross-term *Industry* ^*^Time, which is set to 1 only when both the treatment group and the time of the change are 1. In other cases, the cross-term was set to 0. In this way, the impact of the reforms from CI to UEBMI can be measured.

Other control variables include the nature of the employer, duration of employment, monthly income, gender, age, ethnicity, education, marital status, family size, number of children under 15 yr of age in the household, monthly household income, and monthly household expenditure.

For the robustness check section, a triple difference model or difference-in-difference-in-difference (DDD) was constructed. *Time* represents the time of change and is set to 1 when the year is 2011 and otherwise to 0. *Treat* represents the treatment group and control group in different industries. The construction industry, as the control group, was set at 0. In light of the analysis in the previous section, the wholesale and retail and accommodation and catering industries are more suitable as the treatment group and their value is 1. *The city* represents the treatment group and the control group by city. Chengdu has undergone the policy reform and so comprises the treatment group, with the value 1, while the other cities comprise the control group with the value 0. The cross-term coefficients of *Time, Treat*, and *City* represent the estimates obtained after the triple difference, reflecting the net effect of the policy on the social security participation of migrants in the wholesale and retail and accommodation and catering industries of Chengdu. This was the focus of this study. The triple-difference model was as follows:


(2) $$\begin{array}{l} Insurance_{\text{it}}=\beta_{0}+\beta_{1}Time_{\text{it}}+\beta_{2}\text{Trea}{\text{t}}_{\text{it}}+\beta_{3}City_{\text{it}}\\ \;+\beta_{4}Time_{\text{it}}*\text{Trea}{\text{t}}_{\text{it}}+\beta_{5}Time_{\text{it}}*City_{\text{it}}\\ \;+\beta_{6}\text{Trea}{\text{t}}_{\text{it}}*City_{\text{it}}+\beta_{7}Time_{\text{it}}*\text{Trea}{\text{t}}_{\text{it}}\\ \;*City_{\text{it}}+X_{it}\gamma +\varepsilon_{it} \end{array}$$

## Results

### Impacts of Social Insurance Premium Rates on the Insurance Participation of Migrant Workers

Table 5 presents the estimation results of the DID model. First, there is no significant difference in the participation rates of migrant workers in the construction industry before and after the reform, which means the reform did not change the social security contribution ratio of migrant workers in the construction industry. Second, the migrant workers engaged in wholesale and retail, accommodation and catering, manufacturing, and other industries have significantly higher social insurance participation than those in the construction industry. For construction workers, work injury and medical insurance are extremely important, because they have a higher operating environment risk and are more likely to be accidentally injured or to suffer from various occupational and chronic diseases. Even so, their participation rates are low. Third, the coefficients of the cross-terms are all significantly negative, indicating that the industries affected by the policy have reduced the participation rates of migrant workers due to the increase in the social insurance policy contribution rate (from 20 to nearly 40%). On the one hand, the policy means that the household registration system is no longer an obstacle to the participation of migrant workers in social insurance, allowing migrant workers to enjoy the right to participate in the social insurance on an equal basis and thereby achieving the integration of social insurance and reflecting the equity in this field. On the other hand, it has hindered the popularization of social insurance among the groups of migrant workers. The migrant workers often need social insurance more, but due to their low wages, poor bargaining power, low education, and heavy living burden, higher rates deter them. The main difficulty of current social insurance participation by migrant workers is the low coverage. We should first consider the special characteristics of this group and formulate the social insurance coverage and social insurance payment ratios that are suitable for them and gradually promote integration with the urban employees.

**Table 5:** Impacts of social insurance premium rates on the insurance participation of migrant workers: difference-in-differences (DID) estimates results.

	**Insurance1**	**Insurance2**	**Insurance3**
Time	0.0412	0.0117	−0.0139
	(0.0409)	(0.0371)	(0.0375)
Iindustry2 (Wholesale, Retail and Accommodation)	0.336^***^	0.327^***^	0.361^***^
	(0.0290)	(0.0295)	(0.0298)
Iindustry3 (Manufacturing)	0.348^***^ (0.0280)	0.317^***^ (0.0286)	0.313^***^ (0.0290)
Iindustry4 (Other industries)	0.234^***^ (0.0299)	0.221^***^ (0.0307)	0.237^***^ (0.0310)
Time^*^ind_2	−0.252^***^	−0.182^***^	−0.199^***^
	(0.0408)	(0.0363)	(0.0368)
Time^*^ind_3	−0.0854^**^	−0.108^***^	−0.104^***^
	(0.0435)	(0.0379)	(0.0383)
Time^*^ind_4	−0.118^***^	−0.0494	−0.0455
	(0.0419)	(0.0377)	(0.0382)
Unit1(Private enterprise)	−0.125^***^	−0.181^***^	−0.190^***^
	(0.0219)	(0.0181)	(0.0177)
Unit2(Foreign-owned enterprise)	0.0635	0.0496	0.0359
	(0.0465)	(0.0347)	(0.0341)
Unit3(Small business)	−0.404^***^	−0.469^***^	−0.486^***^
	(0.0221)	(0.0184)	(0.0180)
Unit4(Others)	−0.166^***^	−0.254^***^	−0.269^***^
	(0.0604)	(0.0440)	(0.0440)
Working year	0.00887^***^	0.0116^***^	0.0126^***^
	(0.00155)	(0.00136)	(0.00138)
Ln_income	0.0669^***^	0.0676^***^	0.0771^***^
	(0.0159)	(0.0138)	(0.0139)
Gender	−0.00707	−0.0102	−0.0164
	(0.0121)	(0.0105)	(0.0105)
Age	0.00110	0.000338	0.000672
	(0.000832)	(0.000721)	(0.000723)
Ethnic	−0.0154	−0.0245	−0.00816
	(0.0521)	(0.0474)	(0.0474)
Edu1(Junior)	0.0474^***^	0.0700^***^	0.0777^***^
	(0.0164)	(0.0143)	(0.0145)
Edu2(Secondary)	0.179^***^	0.210^***^	0.218^***^
	(0.0203)	(0.0175)	(0.0176)
Edu2(College degree and above)	0.318^***^	0.339^***^	0.337^***^
	(0.0289)	(0.0240)	(0.0239)
Married	0.0104	0.0101	0.0194
	(0.0300)	(0.0268)	(0.0269)
Family members	−0.00368	0.000208	−0.000863
	(0.00803)	(0.00711)	(0.00713)
Child	−0.0272^**^	−0.0248^**^	−0.0269^***^
	(0.0113)	(0.0101)	(0.0101)
Ln_family income	0.00535	−0.00945	−0.0123
	(0.0121)	(0.0112)	(0.0112)
Ln_family expenditure	0.0132^*^	0.0138^*^	0.0160^**^
	(0.00776)	(0.00736)	(0.00725)
Year fixed effect	Yes	Yes	Yes
Observations	5,890	7,994	8,010
R-squared	0.225	0.228	0.239

*(1) Here, the construction industry/work in government agencies or state-owned, collective, associated enterprises/primary schools, and below are set as the benchmark group*;

(2) ***, **, and * *represent the significant level of 1, 5, and 10% respectively, and the numbers in parentheses are robust SEs. Unless with specification, the following are the same*.

Among other control variables, the social insurance participation rates of migrant populations working in party or government agencies and state-owned/collective/associated enterprises are significantly higher than those in other companies, but these companies or agencies employ less than 10% of the total migrant workers. The private enterprises, individual industrial, and commercial entities have lower compliance with the social insurance contributions, but they comprise more than 80% of the migrant workers. The longer migrant workers stay in the local area, the better they can integrate into it, resulting in higher social insurance participation rates. The higher the salary, the better the job, and the stronger the bargaining power in the company, the more likely workers are to obtain the social insurance paid by their companies. Gender, age, ethnicity, and marital status had no significant effect on social insurance participation. The higher the level of education, the more likely workers are to enter a more formal enterprise and enjoy better treatment; at the same time, their awareness of insurance participation is also stronger. Family size does not affect participation in social insurance, but the greater the number of children in the family, the more the economic cost of the education of children; this renders the family unable to afford the insurance costs. The higher the household expenditure, the better the family benefits, and the higher the social insurance participation rate.

### Impacts of the Social Insurance Premium Rates on the Wages, Working Hours, and Household Expenditures of Migrants

This study also examines the impact of the policy on the wages, working hours, and welfare of migrant workers. As shown in Table 6, after 2011, the wages and benefits of migrant workers in the construction industry significantly improved, and working hours decreased. The wages of workers in the construction industry are higher than those of other industries, which is consistent with common sense; because the construction workers are highly engaged in physical work and face more risks; they should therefore receive higher compensation. However, the household consumption of construction workers is significantly lower than that of other workers, and household consumption is an important indicator of family welfare. The construction workers have a hard time making money and have a heavy burden of living, so their lifestyle is highly constrained. The working hours of the construction workers are similar to those in the manufacturing enterprises, which are higher than those in wholesale, retail, accommodation, and catering. The latter industries have freer and more flexible working hours, and their labor intensity is often lower. The policy did not cause a change in monthly wage income. There may be three possible reasons for this after the social insurance policy contribution rate rises. First, the companies may have maintained the participation status and taken on the higher social insurance fees, choosing to reduce the wages of the migrant workers. Second, the companies may have stopped paying social insurance for employees and subsidized them by increasing wages. Third, the companies may have continued their policy of not paying social insurance and did not change the employee wages. As a result, the overall monthly wages of the migrant workers did not change significantly. However, the weekly working hours of the migrant workers have risen significantly, which may be due to companies passing on the burden of the social insurance costs in disguise by increasing the working hours. Combining these two indicators, the hourly wage of migrant workers has fallen, which has led to a reduction in household consumption. In addition, the decrease in household consumption may also be due to the uncertain expectations of migrant workers about the future employment environment.

**Table 6:** Impacts of the social insurance premium rates on the wages, working hours, and household expenditures of the migrant workers: DID estimation results.

	**Income**	**Work hours**	**Expenditure**
Time	0.502***	−8.752***	0.290***
	(0.0354)	(1.400)	(0.0687)
Iindustry2 (Wholesale, Retail and Accommodation)	−0.209***	−2.304**	0.183***
	(0.0280)	(1.084)	(0.0569)
Iindustry3 (Manufacturing)	−0.175*** (0.0270)	0.783 (1.062)	0.121** (0.0525)
Iindustry4 (Other industries)	−0.261*** (0.0298)	−2.262** (1.119)	0.252*** (0.0643)
Time*ind_2	−0.0530	3.553***	−0.237***
	(0.0343)	(1.308)	(0.0604)
Time*ind_3	0.0248	4.094***	−0.214***
	(0.0349)	(1.346)	(0.0578)
Time*ind_4	0.00302	3.836***	−0.262***
	(0.0363)	(1.353)	(0.0679)
Observations	8,102	7,082	8,102
R-squared	0.558	0.134	0.461

*(1) The first column is monthly salary income; the second column is weekly working hours; the third column is monthly household expenditure. (2) The control variables are consistent with Model 1 and due to space limitations, there is no report here*.

In summary, for migrant workers, an increase in the contribution rate of social insurance policies reduces their participation in social insurance. The wages of migrant workers did not significantly change but the working hours have become longer. Their hourly wages have been reduced to compensate for the increased social insurance burden of enterprises, resulting in a decline in their consumption and welfare.

Since the pre-reform period of the data covers only 2009 and 2010, the effective parallel trend testing could not be performed. To avoid the potential risk that the construction industry may have different trends from other industries, we use a DDD model for testing. CMDS also covers data for other cities that have not experienced the policy change or that have not adopted different policy arrangements for the construction and non-construction companies, which provides us with the opportunity to use the DDD model. The 2009 survey sample includes five cities—Beijing, Shanghai, Shenzhen, Chengdu, and Taiyuan—which share a similar development trend. The four cities other than Chengdu were used as the control group.

Table 7 presents the DDD estimation results of the impact of the social insurance policy contribution rate on the participation by migrant workers. We focus on the coefficients of Time ^*^ Treat ^*^ City. Of the three items measuring the participation of migrant workers, the policy has obvious negative effects on the wholesale, retail, and accommodation industries in Chengdu, which is consistent with the DID results. Therefore, the estimation results of the DDD model show, once again, that an increase in the contribution rate of a social insurance policy will reduce the social insurance participation of migrant workers. At the same time, as shown in Table 8, the policy has no significant impact on the wages and incomes of the migrant workers but reduces the welfare level of the migrant workers. Furthermore, cities similar to Chengdu are selected as the control group to carry out the DDD study. First, in Shenzhen, the migrant workers have always followed the basic social insurance system for urban employees, and the social insurance policy on migrant workers did not change around 2011. Second, Chongqing, a city that shares many similar features with Chengdu, also implemented an integration policy in 2011 but did not implement different social insurance policies for different industries. As shown in Tables 9, 10, a consistent conclusion is obtained after changing the control group. For migrant workers, an increase in the social insurance policy contribution rate will reduce the participation of the migrant workers and their welfare level.

**Table 7:** Impacts of social insurance premium rates on the insurance participation of migrant workers: difference-in-difference-in-difference (DDD) estimates results.

	**Insurance1**	**Insurance2**	**Insurance3**
Time	−0.00629	−0.102***	−0.0944***
	(0.0220)	(0.0202)	(0.0204)
Treat	−0.0210	0.0108	0.0241
	(0.0186)	(0.0187)	(0.0188)
City	−0.157***	−0.133***	−0.125***
	(0.0279)	(0.0288)	(0.0291)
Time*Treat	0.0216	0.0841***	0.0780***
	(0.0217)	(0.0200)	(0.0201)
Time*City	0.0850**	0.121***	0.117***
	(0.0406)	(0.0356)	(0.0360)
Treat*City	0.329***	0.291***	0.310***
	(0.0325)	(0.0335)	(0.0337)
Time*Treat*City	−0.265***	−0.262***	−0.275***
	(0.0461)	(0.0410)	(0.0414)
Year fixed effect	Yes	Yes	Yes
City fixed effect	Yes	Yes	Yes
Observations	11,168	18,174	18,245
R-squared	0.161	0.183	0.194

*(1) The control variables are consistent with Model 1. Due to space limitations, there is no report here*.

**Table 8:** Impacts of the social insurance premium rates on the wages, working hours, and household expenditures of the migrant workers: DDD estimation results.

	**Income**	**Work hours**	**Expenditure**
Time	0.773***	−9.950***	−0.106**
	(0.0182)	(0.640)	(0.0471)
Treat	−0.158***	−1.511***	0.131**
	(0.0170)	(0.574)	(0.0550)
City	−0.0932***	0.511	−0.173**
	(0.0290)	(1.090)	(0.0678)
Time*Treat	0.00732	1.459**	−0.0958*
	(0.0182)	(0.622)	(0.0557)
Time*City	−0.0202	−0.300	0.360***
	(0.0356)	(1.296)	(0.0712)
Treat*City	−0.0602*	1.087	0.0876
	(0.0323)	(1.194)	(0.0814)
Time*Treat*City	−0.0488	1.194	−0.196**
	(0.0395)	(1.440)	(0.0847)
Year fixed effect	Yes	Yes	Yes
City fixed effect	Yes	Yes	Yes
Observations	18,931	16,006	18,894
R-squared	0.547	0.156	0.489

*(1) The control variables are consistent with Model 1. Due to space limitations, there is no report here*.

**Table 9:** Impacts of social insurance premium rates on insurance participation of migrant workers: DDD estimation results using Shenzhen as a control group.

**Panel A**	**Insurance1**	**Insurance2**	**Insurance3**
Time*Treat*City	−0.221**	−0.172**	−0.179**
	(0.0900)	(0.0787)	(0.0802)
Observations	3,312	4,714	4,726
R–squared	0.248	0.242	0.255
Panel B	income	work hours	expenditure
Time*Treat*City	−0.0445	7.929***	−0.490**
	(0.0849)	(2.449)	(0.249)
Year fixed effect	Yes	Yes	Yes
City fixed effect	Yes	Yes	Yes
Observations	4,784	4,168	4,780
R-squared	0.554	0.154	0.501

*(1) The control variables are consistent with Model 1. Due to space limitations, there is no report here*.

**Table 10:** Impacts of social insurance premium rates on the insurance participation of migrant workers: DDD estimation results using Chongqing as a control group.

**Panel A**	**Insurance1**	**Insurance2**	**Insurance3**
Time*Treat*City	−0.212***	−0.148**	−0.157**
	(0.0689)	(0.0720)	(0.0739)
Observations	3,893	6,234	6,235
R-squared	0.154	0.171	0.171
Panel B	income	work hours	expenditure
Time*Treat*City	0.260***	6.169**	−0.969**
	(0.0779)	(2.630)	(0.401)
Year fixed effect	Yes	Yes	Yes
City fixed effect	Yes	Yes	Yes
Observations	6,269	5,061	6,267
R-squared	0.476	0.133	0.363

*(1) The control variables are consistent with Model 1. Due to space limitations, there is no report in this study*.

## Discussion

### Main Findings

The essence of incorporating migrant workers into the urban worker insurance system, in other words, social insurance policy change from CI to UEBMI is to improve the welfare of migrant workers and make them enjoy the same benefits as urban workers. Compared with CI, UEBMI has a higher level of benefits and a higher premium rate. This is the essential reason for the migrant workers to be integrated into the inflow cities. However, in view of the special characteristics of migrant workers, the government should consider the choices of migrant workers when they face low-cost insurance with limited coverage vs. high-cost insurance with higher coverage.

In this study, based on the large sample data of migrant workers in Chengdu from CMDS, we use DID model to evaluate the effects of social insurance policy change from CI to UEBMI and estimate the policy change on the social insurance participation rate, wages, working hours, and household expenditures of the migrant workers. We provided empirical evidence for deepening the social model reform of the migrant workers in China. This study has interesting findings.

We find that the social insurance participation rate of migrant workers was significantly reduced after they were incorporated into the insurance system of urban workers, even though incorporating into the insurance system of urban workers can bring about an obvious improvement in the insurance benefits. Compared with the insurance system of migrant workers (CI), the insurance system of urban workers (UEBMI) has higher premium rates (high-cost) and benefits (high-coverage). That is, the migrant workers prefer low-cost insurance with limited coverage to high-cost insurance with higher coverage. In terms of policy implementation, it does not have a positive impact on migrant workers. Why should such a policy be implemented? The intended goal of the policy is to improve the welfare of migrant workers by combining social insurance; however, what is apparently not taken into account is that the increased level of coverage will also cause an increase in the burden of social insurance for the enterprises. Most small and medium-sized enterprises may transfer the increased burden to their employees because migrant workers do not have bargaining power due to their vulnerable position in the labor market. What is more, given the low wages, low education, and heavy living burden, the migrant workers often choose to increase their current disposable income and give up participating in social insurance when facing a high social insurance premium rate (12, 13).

Meanwhile, the migrant workers have made great contributions to urban development, but they do not enjoy the same benefits as the local urban workers (1–4) because of household registration. The policy change means that the household registration is no longer an obstacle to the participation of migrant workers in social insurance, allowing migrant workers to enjoy the right to participate in social insurance on an equal basis and thereby achieving the integration of social insurance and reflecting the equity in this field. However, the findings demonstrate that the policy change did not have a positive incentive to the popularization of social insurance among the groups of migrant workers but a negative effect.

The unreasonable design of the social insurance pattern is an important reason for this problem; this is mainly reflected in two features. First, the “fragmentation” characteristic of social insurance in China has made it very difficult for migrant workers to transfer their social insurance across regions. Second, the premium rate of social insurance for the migrant workers is high, and the rate of social insurance premiums borne by the migrant workers in most regions is over 10%, which is a high cost for migrant workers to participate in the social insurance. The entrepreneurs in China have complained about the challenging operating environment, with too many regulations and a very high social insurance burden. The urban employee pension scheme requires a contribution equal to 28% of the payroll, 20% from the employer, and 8% from the employee. The 28% contribution rate is above that of the principal Organization for Economic Co-operation and Development (OECD) countries, such as Japan (15.4%), the United States (12.4%), and South Korea (9%). In the 2019 government work report of the 13th National People's Congress, Premier Li Keqiang proposed reducing the basic old-age pension contribution rate of urban employees. The policymakers anticipated that lowering the contribution rate of social insurance could reduce labor costs, promote investment, and create jobs.

### Strengths and Limitations

This study focuses on the social insurance institution design and study the choices of migrant workers when they face different contribution rates and benefits. There are four main contributions: (1) The design of the social insurance system for the migrant workers in Chengdu provides a very unique context for institutional transformation from CI to UEBMI, providing solid evidence for the policymakers to design better social models. This is because direct comparisons of social insurance models across regions can easily be confounded by other factors. Instead, examining the transition process from one model to another in Chengdu allows for clearer identification of the impact of changes in the social insurance models. (2) Based on a nationally representative large sample of survey data for migrant workers, and a double difference (DID) model and triple difference (DDD) model are developed to identify the impact of social insurance patterns on participation and living standards of the migrant workers. This provides an accurate decision basis for the policymakers. (3) This study not only focuses on the impact of social insurance patterns on the incentives of migrant workers to participate in insurance but also examines the possible changes in wages and living standards as a result. Combining changes in the participation rates with changes in wages and living standards allows for a more comprehensive evaluation of the welfare impacts of changes in the social insurance patterns.

However, we studied the impact of social insurance policy premium rate setting on the insurance participation of migrant workers only and paid little attention to the poor portability and fragmentation. Improving the portability and fragmentation of the social insurance system for migrant workers is vital to enhance the welfare of migrant workers. Lacking this discussion made our paper incomplete and this is supposed to be discussed in detail in the future.

### Policy Implications

Therefore, it is necessary to solve the problem of insufficient incentives for migrant workers to participate in social security by formulating the localized social insurance policies.

First, it is necessary to formulate a social security policy premium rate suitable for migrants. After appropriately reducing the employee social security contribution rate, the migrant workers should be included in the employee social security system to achieve uniform contributions and benefits. The current social security contribution rate in China is too high, placing a great burden on labor-intensive enterprises. The small and medium-sized enterprises, where migrant workers are concentrated, are far less able to pay contributions than the large enterprises. Before the social insurance rates are lowered, the inclusion of migrant workers in the urban employee social insurance system would most likely lead to a decrease in their participation rate. Considering the actual situation of the migrant workers, the social insurance premium rate of migrant workers should be reduced to be more practical and to increase their willingness to purchase insurance. The burden on migrant workers can also be reduced by cutting the premium base and by subsidizing or even exempting some social premium costs. In this way, these migrant workers can truly enjoy the benefits of social insurance, thereby increasing the willingness of migrant workers to participate in insurance and maximizing social insurance coverage.

Second, establishing a comprehensive social insurance policy enhances the portability of social security between the regions. This is very important for migrant workers. It is important to gradually integrate CI, UEBMI, and other insurance types into unified insurance, fundamentally changing the fragmented nature of the insurance system. A unified social insurance system with multi-level payment ratios and social insurance benefits that are suitable for different groups of people can truly realize the nationalization and flexibility of the social insurance system.

## Conclusion

This study shows that the migrant workers preferred lower premium rates and that higher premium rates significantly reduced the participation rate of migrant workers in social insurance after being incorporated into the social system of city workers. With an increase in the policy premium rate, the incentive of migrant workers to participate in the social insurance decreased, even if the social insurance benefits correspondingly improved. At the same time, the salaries of migrant workers did not change significantly, but their working hours became longer, and their consumption decreased. Because the government paid little attention to the poor portability and fragmentation of migrant workers, it cannot explore the effects of these policy changes. This is an important direction for some research on the insurance reform of migrant workers in the future.

## Data Availability Statement

Publicly available datasets were analyzed in this study. This data can be found here: China Migrants Dynamic Survey https://chinaldrk.org.cn/wjw/#/home.

## Author Contributions

SZ led and designed the study, led the data collection, analysis, and interpretation. YC contributed to the study design, provided input into the data analysis, and wrote the first draft of the manuscript. YT and MZ contributed to the study design, reviewed the manuscript, and helped the writing of the final draft manuscript. All the authors read and approved the final manuscript.

## Funding

This study was funded by the National Natural Science Foundation of China, Health Care for the Elderly, Medical Expenditure and Savings (71773080), the Full-Time Postdoctoral Research and Development Fund Project of Sichuan University, Research on the Accurate Configuration of Medical Public Services Empowered by Smart Technology in the Post-epidemic Era (skbsh2020-05), and the Independent project of School of Public Administration of Sichuan University, Research on the Accurate Supply of Medical Public Services Empowered by Big Data in the Post-epidemic Era (2020Ziyan-gongguan05).

## Conflict of Interest

The authors declare that the research was conducted in the absence of any commercial or financial relationships that could be construed as a potential conflict of interest.

## Publisher's Note

All claims expressed in this article are solely those of the authors and do not necessarily represent those of their affiliated organizations, or those of the publisher, the editors and the reviewers. Any product that may be evaluated in this article, or claim that may be made by its manufacturer, is not guaranteed or endorsed by the publisher.

## Abbreviations

CI: Urban Employee Basic Medical Insurance
UEBMI: Urban Employee Basic Medical Insurance
DID: Difference-in-difference
DDD: Difference-in-difference-in-difference.
